# *Lactobacillus helveticus* Alleviates Collagen-Induced Arthritis in Rats Through Inflammation Modulation and Gut Microbiota Regulation

**DOI:** 10.3390/nu17233645

**Published:** 2025-11-21

**Authors:** Zhexuan Zhu, Qing Hong, Qixiao Zhai, Jianxin Zhao, Bo Yang, Zhenmin Liu

**Affiliations:** 1 State Key Laboratory of Dairy Biotechnology, Shanghai Engineering Research Center of Dairy Biotechnology, Dairy Research Institute, Bright Dairy & Food Co., Ltd., Shanghai 200436, China; 6230112127@stu.jiangnan.edu.cn (Z.Z.); hongqing1@brightdairy.com (Q.H.); 2 State Key Laboratory of Food Science and Resources, Jiangnan University, Wuxi 214122, China; zhaiqixiao@jiangnan.edu.cn (Q.Z.); zhaojianxin@jiangnan.edu.cn (J.Z.); 3 School of Food Science and Technology, Jiangnan University, Wuxi 214122, China

**Keywords:** *Lactobacillus helveticus*, rheumatoid arthritis, inflammation, gut microbiota, intestinal barrier

## Abstract

**Background**: Rheumatoid arthritis (RA) is a systemic autoimmune disease characterized by chronic synovitis, with growing evidence underscoring the role of gut microbiota dysbiosis and impaired intestinal barrier function in driving inflammation and immune dysregulation. **Methods**: Four strains of *Lactobacillus helveticus* (CCFM1501, DSCAB9M6, CCFM1263, DYNDL451) were evaluated in a collagen-induced arthritis (CIA) rat model. **Results**: *L. helveticus* CCFM1501 exhibited the most pronounced therapeutic benefits. It significantly attenuated paw swelling and synovial hyperplasia and reduced serum levels of total collagen-II-specific IgG and its subclasses (IgG1, IgG2a, IgG2b), pro-inflammatory cytokines (IL-1β, IL-6, IL-17A, TNF-α), and matrix metalloproteinases (MMP-2, MMP-3, MMP-9) while elevating anti-inflammatory IL-10. Moreover, CCFM1501 enhanced intestinal barrier integrity by upregulating tight junction proteins (ZO-1, Occludin, Claudin-1), increased concentrations of short-chain fatty acids (acetic, propionic and butyric acids), and positively restructured gut microbiota composition. **Conclusions**: These findings demonstrate that *L. helveticus* CCFM1501 is associated with the alleviation of CIA, which may be linked to multi-faceted mechanisms including immunomodulation, intestinal barrier repair, SCFA promotion, and microbial homeostasis restoration. These results suggest its potential as a targeted probiotic strategy for RA management and justify further clinical translation.

## 1. Introduction

RA is an autoimmune condition that affects the entire body, in which persistent inflammation of the joint lining (synovitis) is a defining symptom, typically presenting with symmetrical joint involvement, where disease progression shows a strong positive correlation with various autoantibodies [1]. With a global prevalence ranging from 0.24% to 1%, RA affects women at a rate 2–3 times higher than men [2,3], making it a major public health challenge worldwide. Although its exact cause remains elusive, accumulating evidence suggests that RA pathogenesis arises from multifactorial interactions involving genetic susceptibility, environmental triggers—such as smoking and infections—and lifestyle influences [4]. Despite ongoing research, there is still no curative treatment for RA, highlighting the importance of early diagnosis and preventive measures. Timely implementation of disease-modifying therapies remains crucial to curb disease progression. Notably, RA patients display significant gut microbial dysbiosis and impaired intestinal barrier function, which are closely associated with inflammatory responses and immune dysregulation. Treatment with disease-modifying antirheumatic drugs (DMARDs) has been shown to partially restore microbiota composition [5,6], and emerging evidence indicates that DMARDs may exert systemic immunomodulatory effects through structural and functional reshaping of the gut microbial ecosystem [7]. This insight has spurred increasing interest in targeting the gut microbiota—for instance, via probiotic supplementation—as a promising adjunctive therapeutic approach for RA.

In accordance with the consensus definition established by the International Scientific Association for Probiotics and Prebiotics (ISAPP), probiotics are formally defined as “ live microorganisms that, when administered in adequate amounts, confer a health benefit on the host” [8]. Their mechanisms of action are strain-specific and multifunctional, and may include maintaining a healthy microbiota, contributing to the restoration of microbial balance after dysbiosis, and mediating immunomodulation through bioactive metabolites [9]. Accumulating clinical and preclinical evidence supports the therapeutic potential of probiotics in RA, primarily via immunomodulatory pathways [9,10]. For instance, a multi-strain probiotic formulation (containing *Lactobacillus acidophilus* La-14, *L. casei* Lc-11, *Lactococcus lactis* Ll-23, *Bifidobacterium lactis* Bl-04, and *B. bifidum* Bb-06) significantly reduced inflammatory biomarkers such as leukocyte count, TNF-α, and IL-6 in RA patients [11]. *B. longum* subsp. *infantis* B6MNI modulated gut microbiota and fecal 5-HIAA, suppressed Pim-1 expression and immune differentiation, and attenuated joint inflammation and osteoclastogenesis via JAK-STAT3 signaling, thereby delaying RA progression [12]. Similarly, *B. animalis* BD400 altered gut microbiota and histidine metabolism in a CIA rat model, improved intestinal barrier integrity, and suppressed systemic inflammation, resulting in delayed RA pathology [13]. Additionally, *L. helveticus* SBT2171 alleviated CIA progression by inhibiting immune cell proliferation and reducing the production of type II collagen-specific antibodies and IL-6 [14].

*L. helveticus* has demonstrated broad potential as a multifunctional probiotic, contributing to gastrointestinal health, immune responses, and psychobiotic effects. Its high proteolytic activity enables the generation of bioactive peptides with antimicrobial and antioxidant properties [15]. For instance, *L. helveticus* H3 alleviated ETEC-induced diarrhea by suppressing enterotoxin production, restoring fluid balance, promoting SCFAs synthesis, and reducing inflammatory signaling [16]. In a WKY rat model, *L. helveticus* NS8 improved depressive-like behaviors, modulated gut microbiota composition and function, and restored balance to serotonergic and noradrenergic systems [17]. These findings suggest that *L. helveticus* may promote host health through modulations of inflammatory responses and gut microbiota, though these effects are likely multifactorial and strain-specific. To further elucidate this, the current study investigated the prophylactic efficacy of four *L. helveticus* strains on RA. Arthritis severity was assessed via paw swelling thickness and histopathological examination of knee joints, while bone damage was evaluated in rat models. Systemic immune responses were monitored by measuring serum levels of collagen-specific antibodies, pro- and anti-inflammatory cytokines, and matrix metalloproteinases (MMPs). Intestinal barrier integrity was evaluated using qPCR to quantify tight junction protein expression, and changes in gut microbiota composition and diversity were characterized via 16S rRNA sequencing along with SCFAs profiling by GC-MS.

## 2. Materials and Methods

### 2.1. Bacterial Strains Preparation and Culture

The strains *L. helveticus* CCFM1501, DSCAB9M6, CCFM1263, and DYNDL451, preserved at the Culture Collection of Food Microorganisms (CCFM) at Jiangnan University (Wuxi, China), were propagated in MRS broth at 37 °C for 24 h. Bacterial cells were harvested by centrifugation at 6000× *g* for 20 min at 4 °C, washed twice with sterile saline, and resuspended to a working concentration of 3 × 10^9^ CFU/mL. The bacterial suspension was then used for daily oral gavage. Immediately prior to each administration, the bacterial concentration was verified by plating serial dilutions on MRS agar, confirming that the target dose of 3 × 10^9^ CFU/mL was consistently delivered.

### 2.2. Animals and Experimental Design

A total of thirty-six five-week-old specific pathogen-free (SPF) female Wistar rats were obtained from Zhejiang Vital River Laboratory Animal Technology Co., Ltd. (Jiaxing, China). The protocol for animal experiments was approved by the Ethics Committee of Jiangnan University (JN. No. 20240330W080071[154]). All rats were raised under barrier conditions and specific pathogen-free (SPF) conditions, in compliance with the standards of the Laboratory Animal Center of Jiangnan University. The environmental parameters included a temperature of 23 ± 2 °C, humidity of 50 ± 5%, and a 12 h light/dark cycle. The animals were ad libitum fed and were acclimatized for 7 days prior to the start of the formal experiment.

**Figure 1:**
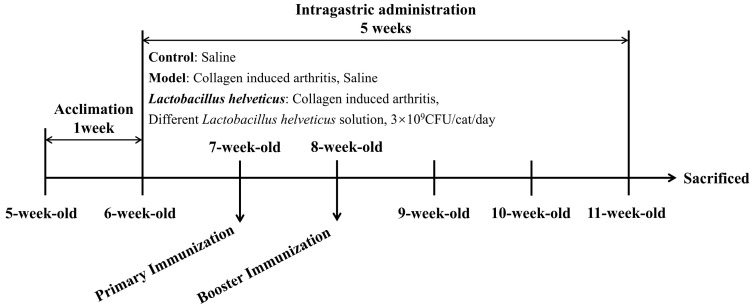
Experimental design.

Following the adaptation period, rats were randomly divided into six groups (*n* = 6) by random number table method: a control group, a model group, and four intervention groups receiving *L. helveticus* strains CCFM1501, DSCAB9M6, CCFM1263, and DYNDL451 (Figure 1). During the experiment, all procedures including disease induction, the thickness of the hind paws measurement, and sample collection were carried out by investigators who were blinded to the group assignment. Throughout the 5-week experiment, the control and model groups received daily oral gavage of 1 mL sterile saline, while the probiotics intervention groups were administered 1 mL of bacterial suspension (3 × 10^9^ CFU/mL) per day. The CIA model, which shares key pathological and immunological features with human RA, was employed in this study. On the first day of weeks 2 and 3, all groups except the control received a subcutaneous injection of 200 μL emulsion—containing 200 μg bovine type II collagen (CII, Chondrex, Redmond, WA, USA) emulsified 1:1 with incomplete Freund’s adjuvant (IFA, Chondrex, Redmond, WA, USA) [18,19] into the tail. The control group received subcutaneous injections of 200 μL sterile saline at the same time points. At the end of week 5, fecal samples were collected for further analysis. Rats were then anesthetized, euthanized, and blood, intestinal tissues and knee joint tissues were harvested for subsequent evaluation.

### 2.3. Assessment of CIA

Throughout the five-week experimental period, rat body weight was monitored on a weekly basis. Following the initial immunization, the thickness of the hind paws was regularly measured using a caliper to quantify paw swelling and assess subsequent changes in arthritis severity.

### 2.4. Knee Joint Histopathology

After dissection, knee joint tissues were collected and fixed in 4% paraformaldehyde for 48 h, prior to decalcification in 10% EDTA for 30 days. The samples were then dehydrated, embedded in paraffin, and sectioned sagittally along the long axis of the knee into 5 µm thick slices. Tissue sections were subjected to hematoxylin and eosin (H&E) staining (Shanghai, China); subsequently, digital histology images were obtained via a Panoramic MIDI slide scanner (3D HISTECH, Budapest, Hungary) [20].

### 2.5. Enzyme-Linked Immunosorbent Assay (ELISA)

Serum was isolated from rat whole blood by centrifugation at 2500× *g* for 15 min. Levels of anti-CII IgG and its subclasses (IgG1, IgG2a, IgG2b) were quantified using commercial ELISA kits (Nanjing SenBeiJia Biological Technology Co., Ltd., Nanjing, China). Simultaneously, serum concentrations of inflammatory cytokines (IL-1β, IL-6, IL-10, IL-17A, and TNF-α) were measured with ELISA kits from Elabscience Biotechnology (Wuhan, China), while MMP-2, MMP-3, and MMP-9 levels were determined using kits supplied by Shanghai Enzyme Linked Biotechnology Co., Ltd. (Shanghai, China). All experimental procedures adhered strictly to the manufacturers’ instructions.

### 2.6. Quantitative Real-Time Polymerase Chain Reaction

Total RNA was extracted from colon tissue stored at −80 °C using a commercial RNA isolation kit (Vazyme Biotech Co., Ltd., Nanjing, China), and its purity and concentration were measured. Reverse transcription was employed to generate complementary DNA (cDNA). Quantitative real-time PCR (qPCR) was conducted with Taq Pro Universal SYBR qPCR Master Mix (Vazyme Biotech Co., Ltd., Nanjing, China) following the manufacturer’s instructions. Relative gene expression were determined by the 2^−ΔΔCt^ method with β-actin as the reference gene, and the corresponding primer sequences were provided in Table 1 [18].

### 2.7. Determination of SCFAs in Feces

The collected rat feces were freeze-dried, and SCFAs were analyzed by gas chromatography–mass spectrometry (GC–MS) according to a previously described method [21]. Briefly, 50 mg of freeze-dried fecal samples was mixed with 500 μL of saturated sodium chloride solution, allowed to stand for 30 min, and homogenized using a tissue disruptor until no visible particles remained. After acidification with 20 μL of sulphuric acid and thoroughly mixing, SCFAs were extracted by adding 800 μL of anhydrous diethyl ether, which was then centrifuged at 16,000× *g* for 15 min. The supernatant was transferred to a 2 mL EP tube containing anhydrous sodium sulphate (0.25%), and centrifuged again under the same conditions to remove residual water. The resulting supernatant was transferred to a GC vial for analysis using a Thermo GC-MS system (Waltham, MA, USA). Separation was achieved on an Rtx-Wax capillary column (30 m × 0.25 mm × 0.25 μm) with helium as the carrier gas at a constant flow rate of 2 mL/min. The injection was performed in split mode (10:1) with a volume of 1 μL at an injector temperature of 240 °C. The oven temperature program was as follows: initial temperature of 100 °C, increased to 140 °C at a rate of 7.5 °C/min, then raised to 200 °C at 60 °C/min and held for 3 min. Mass spectrometric detection was operated in full-scan mode with an ion source temperature of 220 °C. Quantification of individual SCFAs was conducted using the external standard method. All chemical reagents used were procured from Sinopharm Chemical Reagent Co., Ltd. (Shanghai, China) and were of analytical grade.

### 2.8. Analysis of Gut Microbiota

Using the Fast DNA Stool Spin Kit (MP Biomedicals, LLC, Irvine, CA, USA), genomic DNA was obtained from fecal material as per the manufacturer’s guidelines. The V3-V4 hypervariable region of the bacterial 16S rRNA gene was amplified with the universal primers 341F (5′-CCTAYGGGRBGCASCAG-3′) and 806R (5′-GGACTACNNGGGTATCTAAT-3′) [22]. PCR products were subjected to electrophoresis on a 1.5% agarose gel and subsequently purified using a gel extraction kit from BIOMIGA (San Diego, CA, USA). Purified amplicons were sequenced on an Illumina MiSeq platform (San Diego, CA, USA) according to standard protocols, and subsequent 16S rRNA data were processed and analyzed using the QIIME2 pipeline [23]. Following sequencing, the raw reads were processed by removing sample-specific barcodes. Subsequent analysis was performed using the QIIME2 for visualization, the DADA2 for denoising and filtering, and the SILVA database for alignment of Amplicon Sequence Variants (ASVs) from the V3-V4 region.

### 2.9. Statistical Analyses

All data were presented as mean ± standard error of the mean (mean ± SEM), with 95% confidence intervals (95% CI) reported where applicable. Statistical analyses were carried out utilizing SPSS 26.0 and GraphPad Prism 9.5. Intergroup comparisons were determined by one-way analysis of variance (ANOVA), statistical significance was assessed by conducting Duncan’s test after performing ANOVA, adopting a significance threshold of *p* < 0.05.

## 3. Results

### 3.1. Effect of L. helveticus on Body Weight and Hind Paw Thickness of CIA Rats

**Figure 2:**
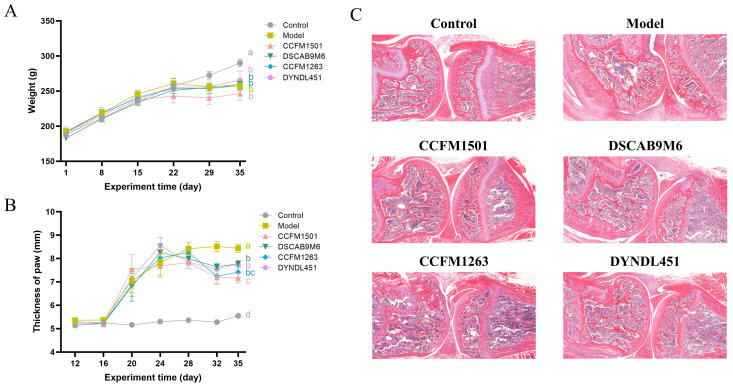
Effects of *L. helveticus* on (**A**) body weight, (**B**) paw thickness, (**C**) representative histopathological sections of knee joints in CIA rats. Data are expressed as the mean ± SEM (*n* = 6 per group). Different lowercase letters indicate statistically significant differences between groups (*p* < 0.05). Tissue sections were stained with hematoxylin and eosin (H&E); scale bar = 500 μm.

Following booster immunization, rats with CIA developed noticeable paw swelling, which impaired mobility and hindered access to food, leading to reduced weight gain or weight loss. With the exception of the control group, all other groups exhibited impaired weight progression (Figure 2A). However, administration of *L. helveticus* strains was associated with a significant alleviation of paw swelling compared to the model group. (Figure 2B).

### 3.2. Effect of L. helveticus on CIA Rat Knee Joint Tissue Pathology Sections

In the model group, cartilage surfaces were extensively covered by proliferative pannus tissue, accompanied by prominent inflammatory cell infiltration, along with evident erosion of both cartilage and subchondral bone. Among the various *L. helveticus*-treated groups, the extent of joint damage varied. Notably, the *L. helveticus* CCFM1501 group showed a marked reduction in tissue hyperplasia, mitigated joint structural damage, and effectively alleviated CIA symptoms (Figure 2C).

### 3.3. Effect of L. helveticus on Anti-CII IgG in the Serum of CIA Rats

**Figure 3:**
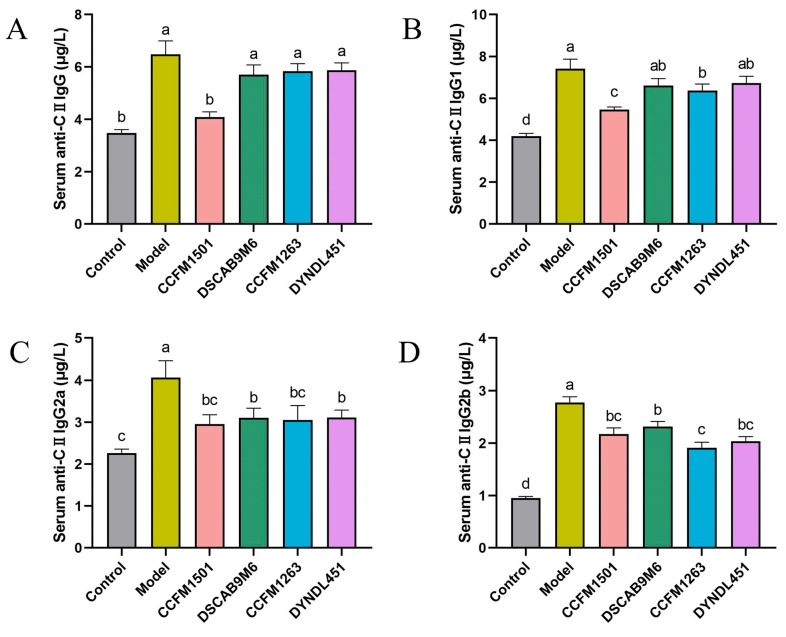
Effects of *L. helveticus* on serum levels of (**A**) total anti-CII IgG; (**B**) anti-CII IgG1; (**C**) anti-CII IgG2a; (**D**) anti-CII IgG2b. Data are expressed as the mean ± SEM (*n* = 6 per group). Different lowercase letters indicate statistically significant differences between groups (*p* < 0.05).

To assess the impact of different treatments on type II collagen-specific immune responses, serum levels of anti-CII IgG and its subclasses (IgG1, IgG2a, and IgG2b) were measured using ELISA. Significantly elevated levels of all four antibodies were observed in the model group compared to the control, confirming successful induction of CIA (Figure 3A–D). *L. helveticus* CCFM1501 group exhibited significantly lower levels of total anti-CII IgG as well as all three subclasses relative to the model group (*p* < 0.05). Similarly, *L. helveticus* CCFM1263 group exhibited significantly lower levels of anti-CII IgG1, anti-CII IgG2a, and anti-CII IgG2b (*p* < 0.05). Both the DSCAB9M6 and DYNDL451 groups also demonstrated decreased levels of anti-CII IgG2a and IgG2b compared to the model group (*p* < 0.05).

### 3.4. Effect of L. helveticus on Inflammatory Factors in the Serum of CIA Rats

**Figure 4:**
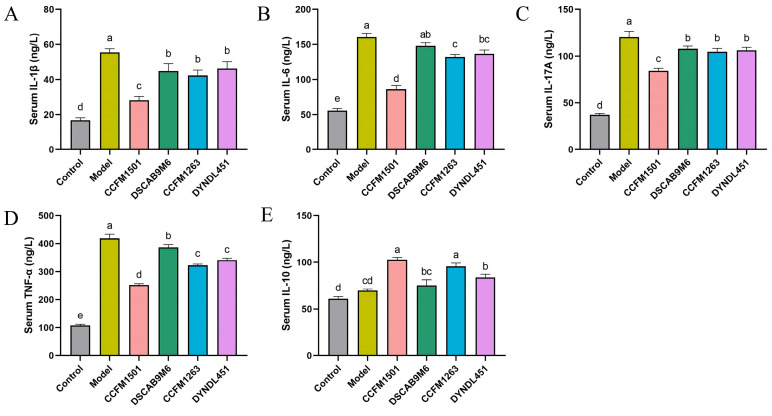
Effects of *L. helveticus* on serum cytokine levels in CIA rats: (**A**) IL-1β; (**B**) IL-6; (**C**) IL-17A; (**D**) TNF-α; (**E**) IL-10. Data are expressed as the mean ± SEM (*n* = 6 per group). Different lowercase letters denote statistically significant differences among groups (*p* < 0.05).

A significant increase in proinflammatory cytokines was observed in the model group compared with the control group (*p* < 0.05) (Figure 4A–E). Administration of *L. helveticus* CCFM1501 significantly reduced serum concentrations of IL-1β, IL-6, IL-17A, and TNF-α, concurrently elevating the anti-inflammatory cytokine IL-10 in CIA rats (*p* < 0.05). Although *L. helveticus* CCFM1263 also reduced pro-inflammatory cytokine levels and elevated IL-10 to some extent, its effects were less pronounced than those of CCFM1501. In contrast, the DSCAB9M6 and DYNDL451 groups had minimal impact on the alteration of serum inflammatory factors.

### 3.5. Effect of L. helveticus on Matrix Metalloproteinases Levels in the Serum of CIA Rats

**Figure 5:**
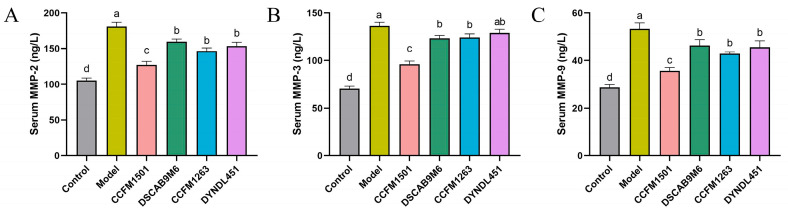
Effects of *L. helveticus* on serum levels of (**A**) MMP-2; (**B**) MMP-3; (**C**) MMP-9 in CIA rats. Data are expressed as the mean ± SEM (*n* = 6 per group). Different lowercase letters indicate statistically significant differences among groups (*p* < 0.05).

Dysregulation of matrix metalloproteinases (MMPs), a key pathological mechanism in RA, directly contributes to progressive joint and periarticular tissue destruction through excessive degradation of the extracellular matrix. Compared to the control group, serum levels of three MMPs were significantly elevated in the model group (Figure 5A–C). Treatment with *L. helveticus* CCFM1501 substantially reduced the MMPs levels (*p* < 0.05). Although CCFM1263 interventions also decreased MMPs concentrations, its effects were less pronouncedly than those of CCFM1501. By comparison, the DSCAB9M6 and DYNDL451 groups exhibited the lowest efficacy in lowering the levels of serum MMPs.

### 3.6. Effects of L. helveticus on Intestinal Barrier Related Protein Genes in CIA Rats

**Figure 6:**
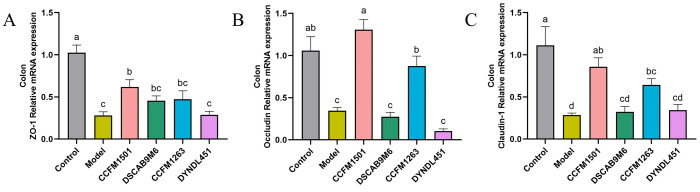
Effects of *L. helveticus* on colon mRNA expression of tight junction proteins in CIA rats: (**A**) ZO-1; (**B**) Occludin; (**C**) Claudin-1. Data are expressed as the mean ± SEM (*n* = 6 per group). Different lowercase letters indicate statistically significant differences among groups (*p* < 0.05).

Compared with the control group, the model group demonstrated a significant downregulation in the mRNA expression of tight junction protein genes (ZO-1, Occludin, and Claudin-1) (*p* < 0.05), indicating compromised intestinal barrier integrity (Figure 6A–C). Administration of *L. helveticus* CCFM1501 significantly upregulated the expression of all three genes compared to the model group (*p* < 0.05), with Occludin and Claudin-1 levels returning close to those of the control group. *L. helveticus* CCFM1263 also moderately enhanced the expression of these tight junction markers, while the remaining experimental groups showed no significant differences relative to the model group (*p* > 0.05).

### 3.7. Effects of L. helveticus on SCFAs in CIA Rats

**Figure 7:**
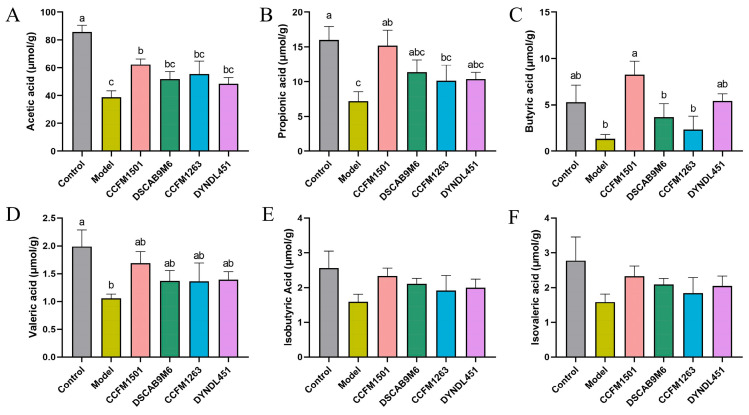
Effects of *L. helveticus* on fecal short-chain fatty acid (SCFA) levels in CIA rats: (**A**) acetic acid; (**B**) propionic acid; (**C**) butyric acid; (**D**) valeric acid; (**E**) isobutyric acid; (**F**) isovaleric acid. Data are expressed as the mean ± SEM (*n* = 5–6 per group). Different lowercase letters indicate statistically significant differences among groups (*p* < 0.05).

A significant reduction in the concentrations of acetate, propionate, butyrate, and valerate was observed in the model group compared to the controls (*p* < 0.05) (Figure 7A–F). Although levels of isobutyrate and isovalerate also decreased in the model group, these differences were not statistically significant (*p* > 0.05). Relative to the model group, *L. helveticus* CCFM1501 significantly elevated the levels of acetate, propionate and butyrate (*p* < 0.05), and also led to a modest elevation in valerate, isobutyrate and isovalerate. No other *L. helveticus* strains showed significant differences in SCFA levels compared to the model group (*p* > 0.05).

### 3.8. Effects of L. helveticus on the Gut Microbiota in CIA Rats

**Figure 8:**
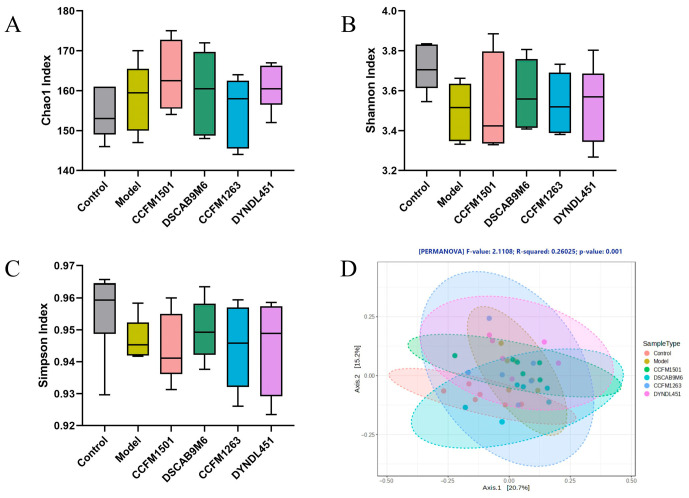
Modulation of *L. helveticus* on gut microbiota in CIA rats: (**A**) Chao1 index, (**B**) Shannon index, (**C**) Simpson index, (**D**) β-diversity PCoA analysis. Data are expressed as the mean ± SEM (*n* = 6 per group).

The α-diversity of gut microbiota, as assessed by Chao1, Shannon, and Simpson indices, showed no significant differences among all groups (*p* > 0.05) (Figure 8A–C). In contrast, principal coordinate analysis (PCoA) of β-diversity revealed a significant separation of the gut microbiota among the six experimental groups (*p* < 0.05), as shown by the distinct clustering in the PCoA plot (Figure 8D).

**Figure 9:**
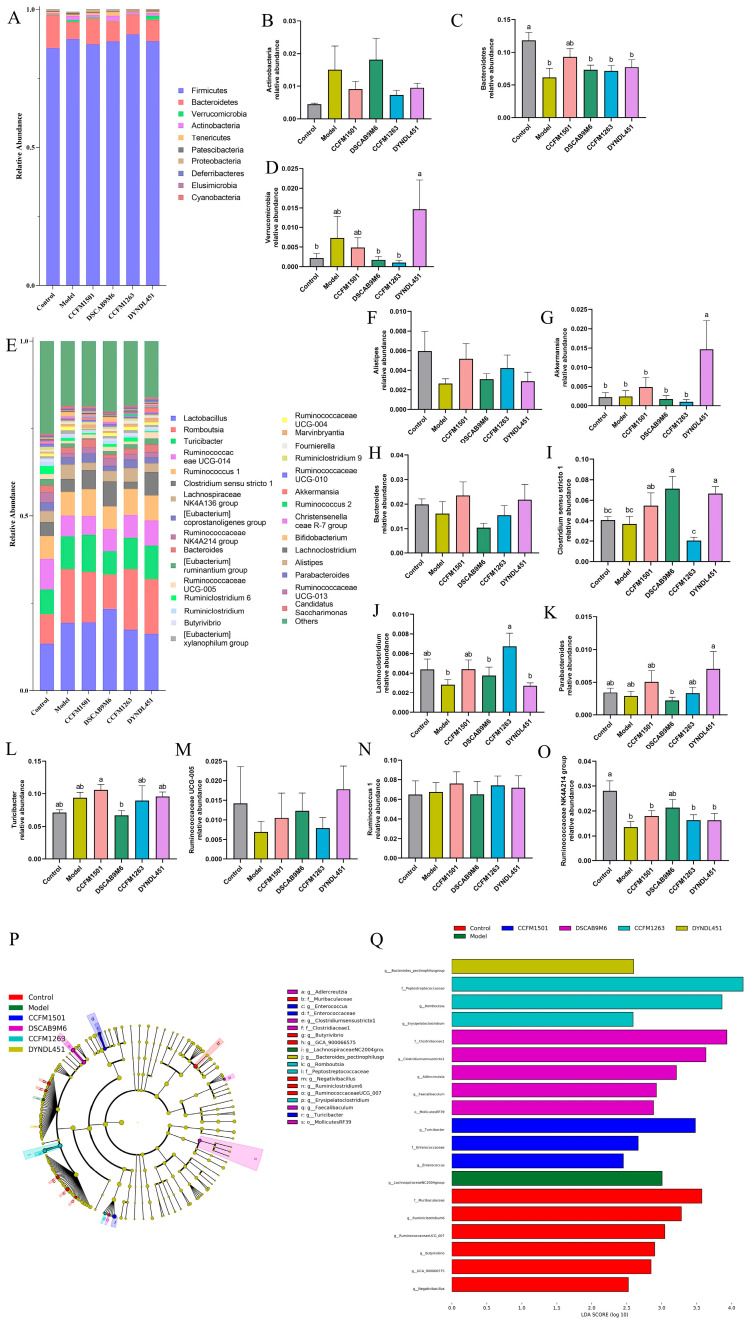
Modulation of *L. helveticus* on gut microbiota in CIA rats. (**A**) microbial composition at the phylum level, (**B**) relative abundance of Actinobacteria, (**C**) relative abundance of Bacteroidetes, (**D**) relative abundance of Verrucomicrobia. (**E**) microbial composition at the genus level, (**F**–**O**) comparison of major genera across groups, (**P**) cladogram of discriminant taxa, (**Q**) LDA distribution histogram (log LDA score > 2.0). Data are expressed as the mean ± SEM (*n* = 6 per group). Different lowercase letters indicate statistically significant differences among groups (*p* < 0.05).

Analysis of gut microbiota composition at the phylum level revealed the following patterns (Figure 9A–D): the model group exhibited a reduction in the relative abundance of Bacteroidetes along with an elevation in the abundances of Verrucomicrobia and Actinobacteria. Treatment with *L. helveticus* CCFM1501 was associated with elevated Bacteroidetes levels and reduced Verrucomicrobia and Actinobacteria. Notably, *L. helveticus* CCFM1263 also decreased the relative abundances of Verrucomicrobia and Actinobacteria, while the DYNDL451 group exhibited reduced Actinobacteria, and the DSCAB9M6 group showed lower Verrucomicrobia.

The LEfSe tool was applied to identify differentially abundant microbial biomarkers across groups, using an LDA score threshold > 2 (Figure 9P,Q). This analysis identified 14 genera showing significant distributional variations among the six experimental groups. Further characterization of the dominant bacterial communities at the genus level revealed several compositional shifts (Figure 9E–O). A significant depletion in *Alistipes*, *Bacteroides*, *Lachnoclostridium*, *Ruminococcaceae UCG-005*, and *Ruminococcaceae NK4A214 group* was observed in the model group in comparison to the control. Treatment with *L. helveticus* CCFM1501 was associated with increased abundance of these taxa, along with elevated levels of *Akkermansia*, *Clostridium sensu stricto 1*, *Parabacteroides*, *Ruminococcus 1*, and *Turicibacter*. Meanwhile, *L. helveticus* DSCAB9M6 increased the relative abundances of *Alistipes*, *Clostridium sensu stricto 1*, *Lachnoclostridium*, *Ruminococcaceae UCG-005*, and *Ruminococcaceae NK4A214 group*; CCFM1263 enriched these four genera except *Clostridium rigidum 1*; while DYNDL451 was associated with higher relative abundances of *Akkermansia*, *Bacteroides*, *Clostridium sensu stricto 1*, *Parabacteroides*, *Ruminococcaceae UCG-005*, and *Ruminococcaceae NK4A214 group*.

**Figure 10:**
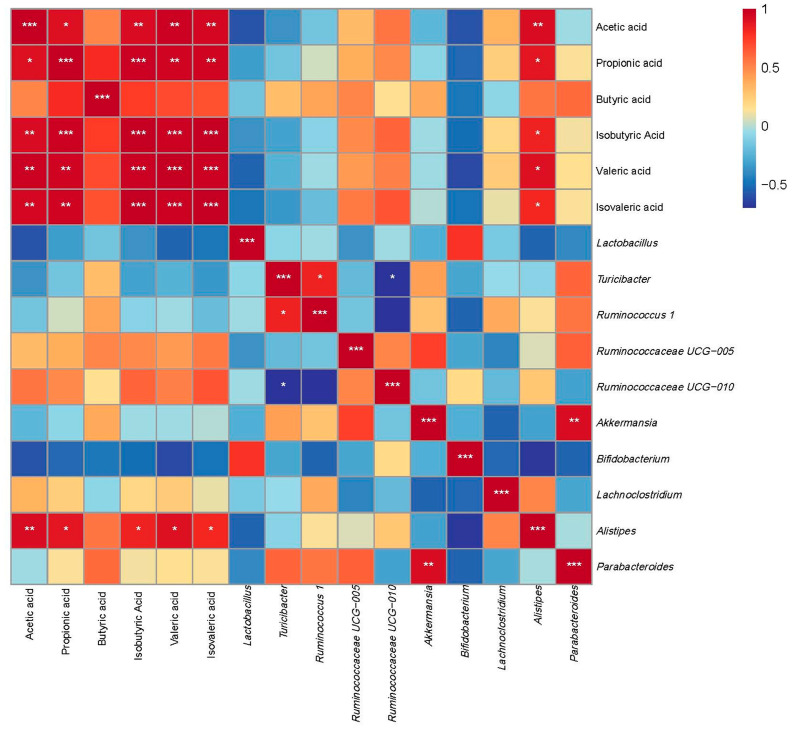
Correlation analysis between gut microbiota composition at the genus level and SCFAs. Data are expressed as the mean ± SEM (*n* = 6 per group). Asterisks indicate a correlation between the two groups; red is a positive correlation, and blue is a negative correlation. * *p* < 0.05, ** *p* < 0.01, and *** *p* < 0.001.

The Pearson correlation analysis was conducted to evaluate the relationships between the relative abundance of selected gut microbiota at the genus level and the concentrations of various SCFAs (Figure 10). *Alistipes* exhibited significant positive correlations with the concentrations of multiple SCFAs, including acetic acid, propionic acid, isobutyric acid, valeric acid, and isovaleric acid (*p* < 0.05).

## 4. Discussion

RA is an autoimmune disorder of unknown etiology marked by inflammatory alterations in synovial tissues, cartilage, and bone. Its progression involves joint pain and swelling, eventually leading to cartilage and bone destruction [24], which can cause functional impairment and substantially reduce patients’ quality of life. Current therapeutic strategies, including NSAIDs, glucocorticoids, DMARDs, and biologics, primarily target inflammatory pathways. Although clinical effective, their non-specific mechanisms and long-term use at high doses may lead to drug resistance and adverse effects such as immunosuppression and organ toxicity [25]. Growing evidence highlights the role of probiotics in RA management, suggesting they may modulate disease progression through immune regulation, intestinal barrier reinforcement, and metabolic homeostasis, supporting their potential as adjunct therapies [26]. A key pathological feature of RA is synovial hyperplasia, which drives the formation of invasive pannus [24]. In this study, treatment with *L. helveticus* CCFM1501 reduced hind paw swelling, suppressed synovial hyperplasia, decreased inflammatory infiltration, and mitigated cartilage erosion and bone destruction. These results are consistent with the joint-protective properties of probiotic intervention and align with previous findings [27].

The CIA model is a well-established experimental system that recapitulates key pathological features of RA, including loss of immune tolerance and autoantibody production [19]. Type II collagen (CII), a major structural component of articular cartilage, acts as a critical autoantigen in RA pathogenesis [28]. Autoantibodies against CII—particularly anti-CII IgG and its subclasses—play a central role in both diagnosis and disease progression, with elevated serum levels often detectable even before clinical symptoms manifest [29]. In this study, serum levels of anti-CII IgG, IgG1, IgG2a, and IgG2b were significantly elevated in CIA rats compared to controls (*p* < 0.05). Oral administration of various *L. helveticus* strains differentially suppressed this increase, with *L. helveticus* CCFM1501 showing the strongest effect, significantly reducing all four antibody subtypes (*p* < 0.05), consistent with earlier findings [14]. Concurrent ELISA measurements revealed alterations in key RA-related mediators, including pro-inflammatory cytokines (IL-6, IL-17A, TNF-α, IL-1β), the anti-inflammatory cytokine IL-10, and matrix metalloproteinases (MMP-2, MMP-3, MMP-9). Among these, TNF-α and IL-6 are recognized as pivotal drivers of RA pathology. These cytokines are considered to drive disease progression through the activation of inflammatory cascades, promotion of synovial hyperplasia, stimulation of osteoclastogenesis, and modulation of immune cell functions, thereby representing critical therapeutic targets in RA [30,31,32]. IL-1β amplifies inflammatory responses through monocyte/macrophage activation, mediates synovial hyperplasia via fibroblast proliferation, and induces cartilage degradation and bone resorption by stimulating chondrocytes and osteoclasts [32]. IL-17, produced locally in RA joints, exacerbates inflammation by triggering synovial cells and chondrocytes to release pro-inflammatory mediators, upregulating MMPs production, and enhancing RANKL expression, thereby accelerating cartilage and bone destruction [33]. In contrast, IL-10 acts as a key anti-inflammatory cytokine that promotes immune tolerance by suppressing innate immune activation, inhibiting pro-inflammatory cytokine release, impairing antigen presentation, and mitigating autoimmunity. Within the RA context, it helps limit inflammation-mediated tissue damage and reduces synovitis and bone erosion [34,35]. Treatment with *L. helveticus* CCFM1501 and CCFM1263 reduced pro-inflammatory cytokine levels while elevating IL-10; notably, CCFM1501 also significantly suppressed MMP-2, MMP-3, and MMP-9, aligning with previous reports [18,36,37]. These results underscore the potential of *L. helveticus* CCFM1501 in alleviating RA-related inflammation and slowing disease progression. Matrix metalloproteinases (MMPs), zinc-dependent endopeptidases, contribute critically to RA pathogenesis by degrading extracellular matrix (ECM) components, disrupting the integrity of synovium, cartilage, and bone [38]. In particular, serum MMP-3 is an established biomarker for evaluating RA disease activity and is widely used in clinical monitoring and prognostic assessment [39,40].

The intestinal barrier, an intricate system of epithelial cells, tight junction proteins, and mucus layers, serves to shield the body from the entry of pathogens and detrimental compounds into the bloodstream. This multi-layered structure plays a vital role in preserving intestinal homeostasis and systemic immune balance, with its integrity being a key determinant of overall health [41]. Increased intestinal permeability is recognized as a critical factor in the pathogenesis of RA. Tight junctions (TJs), which include transmembrane proteins such as Claudins, Occludin, ZO, and JAM, along with peripheral scaffolding proteins [18], play a central role in preserving barrier function and intestinal homeostasis [42]. Dysfunction of TJs compromises barrier integrity, leading to the leakage of intestinal contents, formation of a pro-inflammatory milieu, and recruitment and activation of autoreactive Th17 and effector T cells, thereby exacerbating RA [41]. In this study, treatment with *L. helveticus* CCFM1501 was associated with a significant elevation in the mRNA expression levels of ZO-1, Claudin-1, and Occludin in the colon, suggesting a potential enhancement in intestinal barrier integrity. Short-chain fatty acids (SCFAs), which comprise acetate, propionate, and butyrate, are primarily generated via microbial fermentation of dietary fibers and resistant starch in the colon [43]. They enter systemic circulation via the gut-blood barrier and exert modulatory effects on distal joints and immune cells [41]. SCFAs exhibit antimicrobial and anti-inflammatory properties that contribute to the amelioration of RA pathology. They modulate RA through both osteo-regulatory and immuno-regulatory mechanisms [44]. For example, butyrate inhibits osteoclastogenesis by suppressing histone deacetylase 2 (HDAC2) activity in osteoclasts [45], and helps restore immune homeostasis by rebalancing Th17/Treg and Tfr/Tfh cell ratios while expanding anti-inflammatory regulatory B cells (Bregs), thereby balancing pro- and anti-inflammatory responses [46]. RA patients show markedly reduced levels of microbiota-derived SCFAs, and supplementation with acetate, propionate, and butyrate has been shown to alleviate arthritis in CIA models [47]. SCFAs also enhance intestinal barrier function by activating MUC2 expression to strengthen the mucus layer and modulating the expression and distribution of tight junction proteins, collectively improving gut barrier integrity [48]. In this study, *L. helveticus* CCFM1501 significantly increased levels of acetate, propionate and butyrate in CIA rats. These changes suggest a potential link between the alleviation of RA symptoms and the restoration of SCFAs homeostasis, which aligns with the observed upregulation of tight junction proteins and underscores the multi-faceted beneficial effects of this probiotic strain.

In healthy individuals, gut microbiota demonstrates substantial species richness and stability, forming a dynamic and balanced ecosystem that plays a key role in sustaining intestinal and immune homeostasis [49]. Current research on RA highlights significant gut microbiota alterations, typically marked by a reduction in beneficial bacteria and an increase in pathogenic taxa. This dysbiosis is closely associated with immune dysregulation and chronic inflammation, contributing to RA progression [41]. Notable compositional differences in gut microbiota of RA patients compared to healthy controls have been linked to clinical symptoms [50]. In this study, although oral administration of *L. helveticus* did not significantly alter overall microbial diversity, different strains induced distinct compositional shifts in the gut microbiota of rats. In agreement with reports in RA patients and CIA models [51,52,53], reduced abundance of Bacteroidetes and increased levels of Actinobacteria and Verrucomicrobia were observed. Treatment with *L. helveticus* CCFM1501 reversed these dysbiotic trends and partially restored a healthier microbial profile. Specifically, *L. helveticus* CCFM1501 significantly increased the abundance of multiple beneficial genera, including *Alistipes*, *Bacteroides*, *Lachnoclostridium*, *Ruminococcaceae UCG-005*, *Ruminococcaceae NK4A214*, *Akkermansia*, *Clostridium sensu stricto 1*, *Parabacteroides*, *Ruminococcus 1*, and *Turicibacter*. For example, *Alistipes*—which is often reduced in inflammatory conditions such as advanced non-alcoholic fatty liver disease (NAFLD)—correlates positively with SCFA levels, suggesting its potential role in mitigating disease through SCFA production [54]. Similarly, *Bacteroides* contribute to host health via beneficial metabolites, including SCFAs and secondary bile acids [55]. *Lachnoclostridium*, a butyrate producer, has been associated with potential protective effects in RA patients mechanisms [56], while *Akkermansia* supports immune regulation, metabolic health, and barrier function [57]. *Parabacteroides*, particularly *P. distasonis,* may benefit RA through immunomodulation, SCFAs production, and barrier repair [53]. The modulation of these key taxa by *L. helveticus* CCFM1501 was correlated with a trend toward microbial balance reestablishment, a reduction in inflammation linked to dysbiosis, and an alleviation of RA symptoms.

This study has several limitations that should be acknowledged. First, regarding the animal model, the exclusive use of female rats—while justified by the higher disease prevalence in females—limits the generalizability of our findings to males and precludes insights into sex-specific pathophysiology. Additionally, the uncontrolled estrous cycle may confound immune and microbial measurements. Second, methodologically, the lack of baseline microbiota profiling complicates the interpretation of treatment-induced changes, and the necessary use of oral gavage may introduce transient stress. Finally, causal inference is limited by the unverified colonization of the probiotic strain and the absence of functional analyses to mechanistically interpret the observed microbial shifts.

A logical progression for future research is proposed. Initial efforts should employ fecal microbiota transplantation (FMT) to definitively establish causal relationships. Subsequent investigations would benefit from integrated multi-omics analyses (metagenomics and metabolomics) to delineate functional mechanisms and key metabolites, complemented by co-occurrence network analysis and functional prediction tools (e.g., PICRUSt2) to link microbial ecology to host physiology. Furthermore, the inclusion of both sexes in subsequent studies is essential to evaluate translational relevance. Collectively, these steps are critical to inform the design of clinical trials for *L. helveticus* CCFM1501 in rheumatoid arthritis patients.

## 5. Conclusions

A comparative analysis of four *L. helveticus* strains revealed distinct strain-specific effects in alleviating RA. Among them, *L. helveticus* CCFM1501 exhibited the most pronounced therapeutic effects, which coincided with multi-faceted mechanisms including modulation of inflammatory cytokines, reduction in serum type II collagen-specific antibodies and MMPs levels, enhancement in intestinal barrier function, promotion of SCFAs production, and restoration of gut microbiota composition. These results suggest that gut dysbiosis and compromised intestinal barrier integrity may play instrumental roles in RA pathogenesis and that *L. helveticus* CCFM1501 likely attenuates arthritis by modulating this coordinated gut-joint axis. The findings are consistent with the potential of *L. helveticus* CCFM1501 as a probiotic candidate for RA management and support the case for further clinical evaluation. However, the precise molecular and immunological mechanisms through which CCFM1501 exerts its effects remain incompletely understood and warrant deeper investigation in future studies. Building on our findings, we propose a testable hypothesis that CCFM1501 alleviates RA via a gut microbiota-metabolite axis. Future studies employing FMT and targeted metabolite interventions are warranted to formally establish this causal chain.

## Figures and Tables

**Table 1:** The primers for this study.

Gene	Forward (5′-3′)	Reverse (5′-3′)
*ZO-1*	GGCGTTCTAGAAGATAGCC	GAAATCTACATTGTTCACCCTG
*Claudin-1*	TCTGAATAGTACTTTGCAGGC	GTGGACACAAAGATTGCGA
*Occludin*	ACTATGAAACCGACTACACGA	TGATAGGTGGATATTCCCTGAG
*β-actin*	CTTCCTGGGTATGGAATCCT	TCTTTACGGATGTCAACGTC

## Data Availability

The original contributions presented in this study are included in the article. Further inquiries can be directed to the corresponding authors.

## Abbreviations

The following abbreviations are used in this manuscript:

RA	Rheumatoid arthritis
CIA	Collagen-induced arthritis
CCFM	Culture Collection of Food Microorganisms
DMARDs	Disease-Modifying Antirheumatic Drugs
ISAPP	International Scientific Association for Probiotics and Prebiotics
MRS	Man Rogosa Sharpe Medium
SPF	Specific-pathogen-free
IL-1β	Interleukin-1β
IL-6	Interleukin-6
IL-17A	Interleukin-17A
TNF-α	Tumor necrosis factor-α
IL-10	Interleukin-10
MMP-2	Matrix Metallopeptidase-2
MMP-3	Matrix Metallopeptidase-3
MMP-9	Matrix Metallopeptidase-9
ZO-1	Zona occludens 1
SCFAs	Short-chain fatty acids
